# Proceedings of the Medical Society at the Meeting in Ottawa in May Last

**Published:** 1857-09

**Authors:** 


*Death of Dr. Marshall Hall.*

We learn from the London *Lancet,* that this most distinguished Physiologist and Physician died at Brighton, England, on the 11th of August ult., aged 67 years. But few men have attained a higher or more deserved reputation than Dr. MARSHALL HALL, and his death will be universally regretted.

